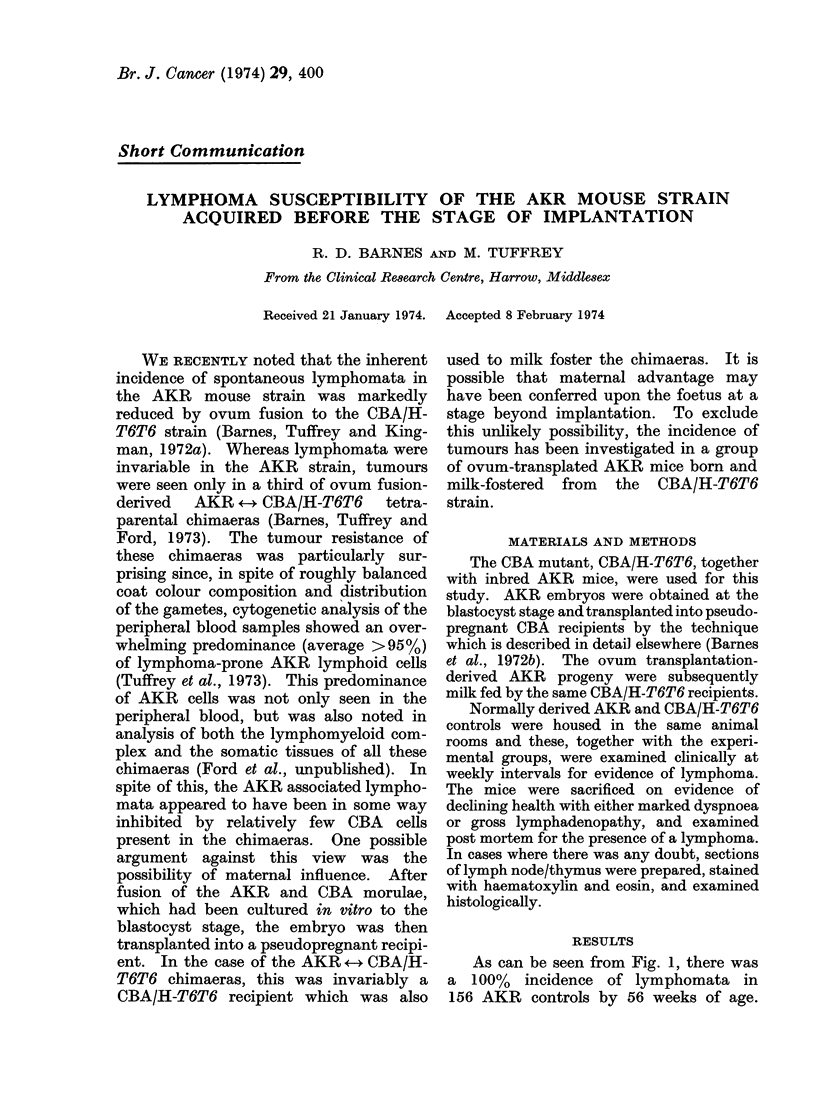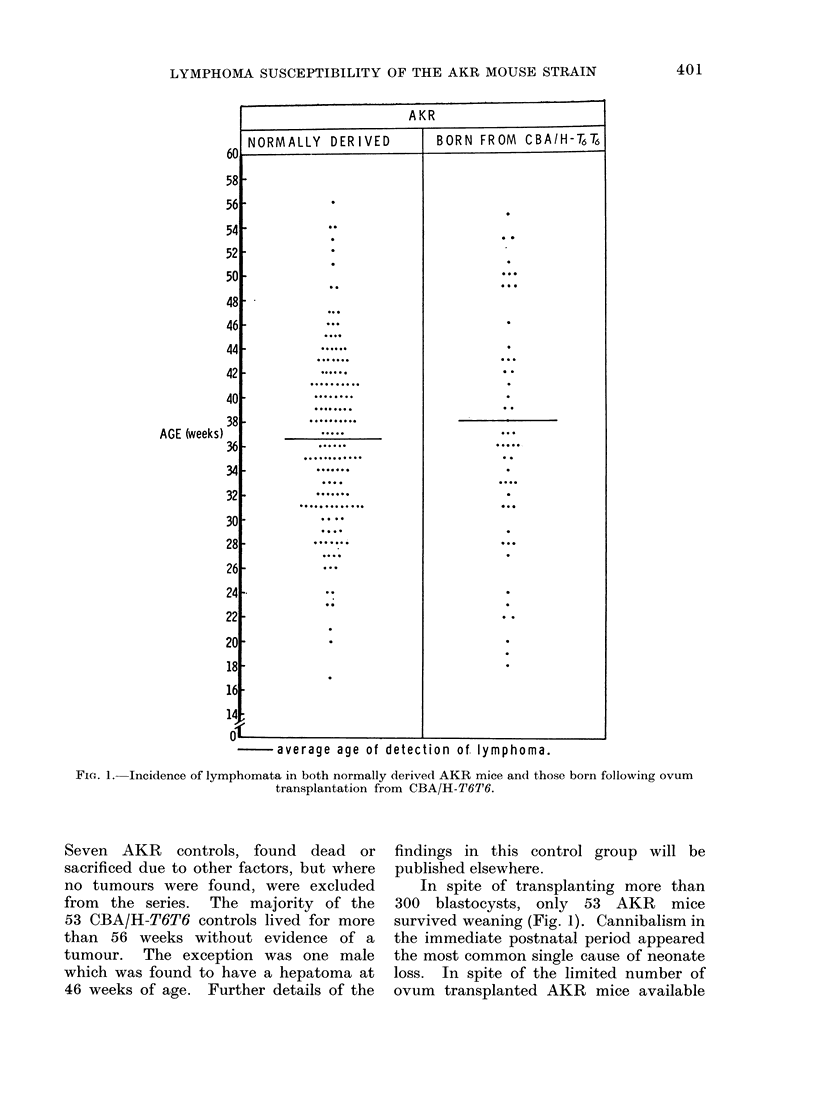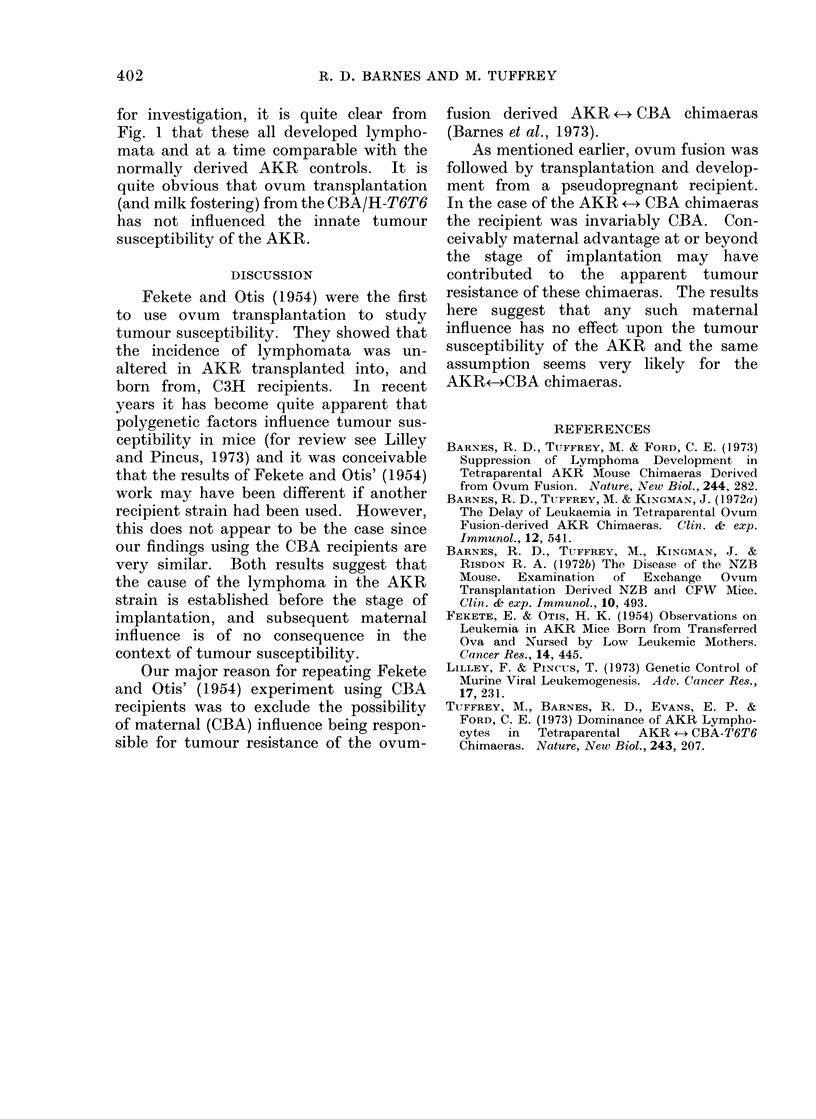# Lymphoma susceptibility of the AKR mouse strain acquired before the stage of implantation.

**DOI:** 10.1038/bjc.1974.87

**Published:** 1974-05

**Authors:** R. D. Barnes, M. Tuffrey


*Br. J. Cancer* (1974) **29**, 400

### *Short Communication*

### LYMPHOMA SUSCEPTIBILITY OF THE AKR MOUSE STRAIN ACQUIRED BEFORE THE STAGE OF IMPLANTATION

R. D. BARNES AND M. TUFFREY

*From the Clinical Research Centre, Harrow, Middlesex*

Received 21 January 1974. Accepted 8 February 1974

WE RECENTLY noted that the inherent incidence of spontaneous lymphomata in the AKR mouse strain was markedly reduced by ovum fusion to the CBA/H-*T6T6* strain (Barnes, Tuffrey and Kingman, 1972*a*). Whereas lymphomata were invariable in the AKR strain, tumours were seen only in a third of ovum fusion-derived AKR ↔ CBA/H-*T6T6* tetraparental chimaeras (Barnes, Tuffrey and Ford, 1973). The tumour resistance of these chimaeras was particularly surprising since, in spite of roughly balanced coat colour composition and distribution of the gametes, cytogenetic analysis of the peripheral blood samples showed an overwhelming predominance (average >95%) of lymphoma-prone AKR lymphoid cells (Tuffrey *et al.*, 1973). This predominance of AKR cells was not only seen in the peripheral blood, but was also noted in analysis of both the lymphomyeloid complex and the somatic tissues of all these chimaeras (Ford *et al.*, unpublished). In spite of this, the AKR associated lymphomata appeared to have been in some way inhibited by relatively few CBA cells present in the chimaeras. One possible argument against this view was the possibility of maternal influence. After fusion of the AKR and CBA morulae, which had been cultured *in vitro* to the blastocyst stage, the embryo was then transplanted into a pseudopregnant recipient. In the case of the AKR ↔ CBA/H-*T6T6* chimaeras, this was invariably a CBA/H-*T6T6* recipient which was also used to milk foster the chimaeras. It is possible that maternal advantage may have been conferred upon the foetus at a stage beyond implantation. To exclude this unlikely possibility, the incidence of tumours has been investigated in a group of ovum-transplated AKR mice born and milk-fostered from the CBA/H-*T6T6* strain.

### MATERIALS AND METHODS

The CBA mutant, CBA/H-*T6T6*, together with inbred AKR mice, were used for this study. AKR embryos were obtained at the blastocyst stage and transplanted into pseudopregnant CBA recipients by the technique which is described in detail elsewhere (Barnes *et al.*, 1972*b*). The ovum transplantation-derived AKR progeny were subsequently milk fed by the same CBA/H-*T6T6* recipients.

Normally derived AKR and CBA/H-*T6T6* controls were housed in the same animal rooms and these, together with the experimental groups, were examined clinically at weekly intervals for evidence of lymphoma. The mice were sacrificed on evidence of declining health with either marked dyspnoea or gross lymphadenopathy, and examined post mortem for the presence of a lymphoma. In cases where there was any doubt, sections of lymph node/thymus were prepared, stained with haematoxylin and eosin, and examined histologically.

### RESULTS

As can be seen from Fig. 1, there was a 100% incidence of lymphomata in 156 AKR controls by 56 weeks of age.

Fig. 1.—Incidence of lymphomata in both normally derived AKR mice and those born following ovum transplantation from CBA/H-*T6T6*.

Seven AKR controls, found dead or sacrificed due to other factors, but where no tumours were found, were excluded from the series. The majority of the 53 CBA/H-*T6T6* controls lived for more than 56 weeks without evidence of a tumour. The exception was one male which was found to have a hepatoma at 46 weeks of age. Further details of the findings in this control group will be published elsewhere.

In spite of transplanting more than 300 blastocysts, only 53 AKR mice survived weaning (Fig. 1). Cannibalism in the immediate postnatal period appeared the most common single cause of neonate loss. In spite of the limited number of ovum transplanted AKR mice available for investigation, it is quite clear from Fig. 1 that these all developed lymphomata and at a time comparable with the normally derived AKR controls. It is quite obvious that ovum transplantation (and milk fostering) from the CBA/H-*T6T6* has not influenced the innate tumour susceptibility of the AKR.

### DISCUSSION

Fekete and Otis (1954) were the first to use ovum transplantation to study tumour susceptibility. They showed that the incidence of lymphomata was unaltered in AKR transplanted into, and born from, C3H recipients. In recent years it has become quite apparent that polygenetic factors influence tumour susceptibility in mice (for review see Lilley and Pincus, 1973) and it was conceivable that the results of Fekete and Otis' (1954) work may have been different if another recipient strain had been used. However, this does not appear to be the case since our findings using the CBA recipients are very similar. Both results suggest that the cause of the lymphoma in the AKR strain is established before the stage of implantation, and subsequent maternal influence is of no consequence in the context of tumour susceptibility.

Our major reason for repeating Fekete and Otis' (1954) experiment using CBA recipients was to exclude the possibility of maternal (CBA) influence being responsible for tumour resistance of the ovum-fusion derived AKR ↔ CBA chimaeras (Barnes *et al.*, 1973).

As mentioned earlier, ovum fusion was followed by transplantation and development from a pseudopregnant recipient. In the case of the AKR ↔ CBA chimaeras the recipient was invariably CBA. Conceivably maternal advantage at or beyond the stage of implantation may have contributed to the apparent tumour resistance of these chimaeras. The results here suggest that any such maternal influence has no effect upon the tumour susceptibility of the AKR and the same assumption seems very likely for the AKR↔CBA chimaeras.

### REFERENCES

Barnes, R. D., Tuffrey, M. & Ford, C. E. (1973) Suppression of Lymphoma Development in Tetraparental AKR Mouse Chimaeras Derived from Ovum Fusion. *Nature, New Biol.*, **244**, 282.

Barnes, R. D., Tuffrey, M. & Kingman, J. (1972*a*) The Delay of Leukaemia in Tetraparental Ovum Fusion-derived AKR Chimaeras. *Clin. & exp. Immunol.*, **12**, 541.

Barnes, R. D., Tuffrey, M., Kingman, J. & Risdon R. A. (1972*b*) The Disease of the NZB Mouse. Examination of Exchange Ovum Transplantation Derived NZB and CFW Mice. *Clin. & exp. Immunol.*, **10**, 493.

Fekete, E. & Otis, H. K. (1954) Observations on Leukemia in AKR Mice Born from Transferred Ova and Nursed by Low Leukemic Mothers. *Cancer Res.*, **14**, 445.

Lilley, F. & Pincus, T. (1973) Genetic Control of Murine Viral Leukemogenesis. *Adv. Cancer Res.*, **17**, 231.

Tuffrey, M., Barnes, R. D., Evans, E. P. & Ford, C. E. (1973) Dominance of AKR Lymphocytes in Tetraparental AKR ↔ CBA-*T6T6* Chimaeras. *Nature, New Biol.*, **243**, 207.